# Impacts of Rotational Grazing on Soil Carbon in Native Grass-Based Pastures in Southern Australia

**DOI:** 10.1371/journal.pone.0136157

**Published:** 2015-08-18

**Authors:** Jonathan Sanderman, Jodie Reseigh, Michael Wurst, Mary-Anne Young, Jenet Austin

**Affiliations:** 1 CSIRO Sustainable Agriculture Flagship, Urrbrae, South Australia, Australia; 2 Rural Solutions SA, Primary Industries and Regions South Australia, Adelaide, South Australia, Australia; 3 CSIRO Land and Water Flagship, Black Mountain, Australian Capital Territory, Australia; USDA-ARS, UNITED STATES

## Abstract

Rotational grazing management strategies have been promoted as a way to improve the sustainability of native grass-based pasture systems. From disturbance ecology theory, rotational grazing relative to continuous grazing can increase pasture productivity by allowing vegetation to recover after short intense grazing periods. This project sought to assess whether soil organic carbon (SOC) stocks would also increase with adoption of rotational grazing management. Twelve pairs of rotationally and continuously grazed paddocks were sampled across a rainfall gradient in South Australia. Pasture productivity approximated as the normalized difference vegetation index (NDVI) was on average no different between management categories, but when the data from all sites were aggregated as log response ratios (rotational/continuous) a significant positive trend of increasing NDVI under rotational grazing relative to continuous grazing was found (R^2^ = 0.52). Mean SOC stocks (0–30 cm) were 48.3 Mg C ha^-1^ with a range of 20–80 Mg C ha^-1^ across the study area with no differences between grazing management categories. SOC stocks were well correlated with rainfall and temperature (multiple linear regression R^2^ = 0.61). After removing the influence of climate on SOC stocks, the management variables, rest periods, stocking rate and grazing days, were found to be significantly correlated with SOC, explaining 22% of the variance in SOC, but there were still no clear differences in SOC stocks at paired sites. We suggest three reasons for the lack of SOC response. First, changes in plant productivity and turnover in low-medium rainfall regions due to changes in grazing management are small and slow, so we would only expect at best small incremental changes in SOC stocks. This is compounded by the inherent variability within and between paddocks making detection of a small real change difficult on short timescales. Lastly, the management data suggests that there is a gradation in implementation of rotational grazing and the use of two fixed categories (i.e. rotational v. continuous) may not be the most appropriate method of comparing diverse management styles.

## Introduction

Recently, there has been much interest in agricultural management for maintaining or enhancing soil organic carbon (SOC) levels. Globally, it has been estimated that agricultural soils have lost 42–78 Pg C relative to their pre-agricultural state [1]. This transfer of SOC to the atmosphere is a major perturbation to the global carbon cycle [2], but also represents an opportunity for managing current greenhouse gas emissions through carbon sequestration [3]. Importantly, this loss of SOC has negatively affected soil health and increases our reliance on inorganic fertilizers to maintain crop productivity. A large number of soil functions that are critical for crop and pasture production, including nutrient and pH buffering, water retention, soil structural stability, and higher agronomic efficiency with respect to fertilizer inputs are all positively associated with greater SOC levels [4], [5]. Halting or reversing the decline in SOC in agricultural soils is seen as a win-win policy because of the dual benefits to soil sustainability/production and greenhouse gas abatement. Due to this fact, many nations are actively promoting management strategies that have the potential to sequester carbon. Rotational grazing is one such carbon management strategy that has an additional benefit in that it is seen as being consistent with the protection of the natural environment and improves resilience to the impacts of climate change [6].

Rotational grazing, defined as where a paddock is not stocked continuously but grazed and rested regularly either on a set calendar schedule or intermittently as needed [7], when compared to continuous grazing, defined as where a paddock is stocked continuously at generally consistent stock density whether or not it is with the same animals [7], is generally thought to have a number of production and biodiversity benefits. The benefits of rotational grazing include even grazing pressure [8], [9], [10]; reduced herbivore selectivity and selection of palatable species [8], [11]; enhanced flowering, growth and survival of plant species [12], [13]; improved pasture utilization [14]; maintenance of pasture cover [15], [16], [17], [18]; higher perennial grass content [19], [20]; increased herbage production [18]; increased perennial basal area [16], [20], [11]; reduced soil erosion [17] and improved animal production [16]. However, Briske et al. [21] stressed that the experimental evidence on a whole is decidedly mixed with many more studies reporting no demonstrable benefit of rotational grazing.

Many of the reported benefits of rotational grazing especially those related to pasture production can translate into increases in SOC in rotationally grazed paddocks relative to continuously grazed paddocks because, all else being equal, greater organic matter inputs to the soil will lead to greater SOC levels [22]. Persistent groundcover resulting in decreased erosion will have a two-fold SOC benefit–elimination of a direct loss pathway via eroded topsoil and maintenance of improved soil structure [23]. There is also some evidence that belowground microbial activity can be enhanced under rotational grazing [24] which may result in greater stabilization of organic matter [25]. Clearly, there is evidence to support improvement of SOC levels with adoption of rotational grazing.

However, the field evidence for carbon sequestration is inconsistent. In a global meta-analysis, Conant et al. [26] suggested that for most management techniques in grasslands, such as fertilization and improvement of grazing management, average relative gains in SOC in the 0–0.3 m horizon are of the order of 1–2% per year, corresponding to average sequestration rates of 0.3 Mg C ha^-1^ yr^-1^. However, the variance around that mean value was large and in many individual studies the data were insignificantly different between management categories [27], [28], [29]. Additionally, there is evidence that increasing grazing intensity may have different effects in C3 versus C4 dominated pastures [30].

In the current study, we sought to better understand if rotational grazing relative to continuous grazing of remnant native grass-based pastures resulted in increases in SOC levels and if there were specific management practices which were more effective than others in increasing SOC.

## Methods

### 2.1. Field sites

Approximately 20 landowners were identified as having adopted rotational grazing practices over the past 5–15 years in the upper and mid-north of South Australia, a region covering about 200 km in latitude and 60 in longitude. Grazed pastures are generally confined to the non-arable upper slopes of the hilly ranges that tend to be rocky, with shallower soils and too steep to traverse with machinery. Pasture composition is generally dominated by native perennial grasses *Austrostipa* species (Spear grass) and *Rytidosperma* species (Wallby grass) and annual grasses *Avena barbata* (wild oats), *Vulpia* species (Sliver grass), *Bromus* species (Brome grass) and a range of herbs and forbs (nomenclature follows [31]). However, the composition is largely dependent on past management, particularly grazing management, fertiliser application and any history of cultivation. Rainfall varies across this region from 310 to 570 mm yr^-1^ (winter dominant) with mean winter temperatures of 10.5–13.5°C and summer temperatures of 20.1–21.9°C. Mean maxima temperatures tend to increase from south to north and minima temperature decreases, but temperatures are also influenced by elevation. Soils were classified using the Australian Soil Classification [32] as Red Chromosols; however, Calcarosols were encountered at some sites. In the USDA-NRCS classification [33], these soils would be Rhodoxeralfs and Haplocalcids.

**Fig 1:**
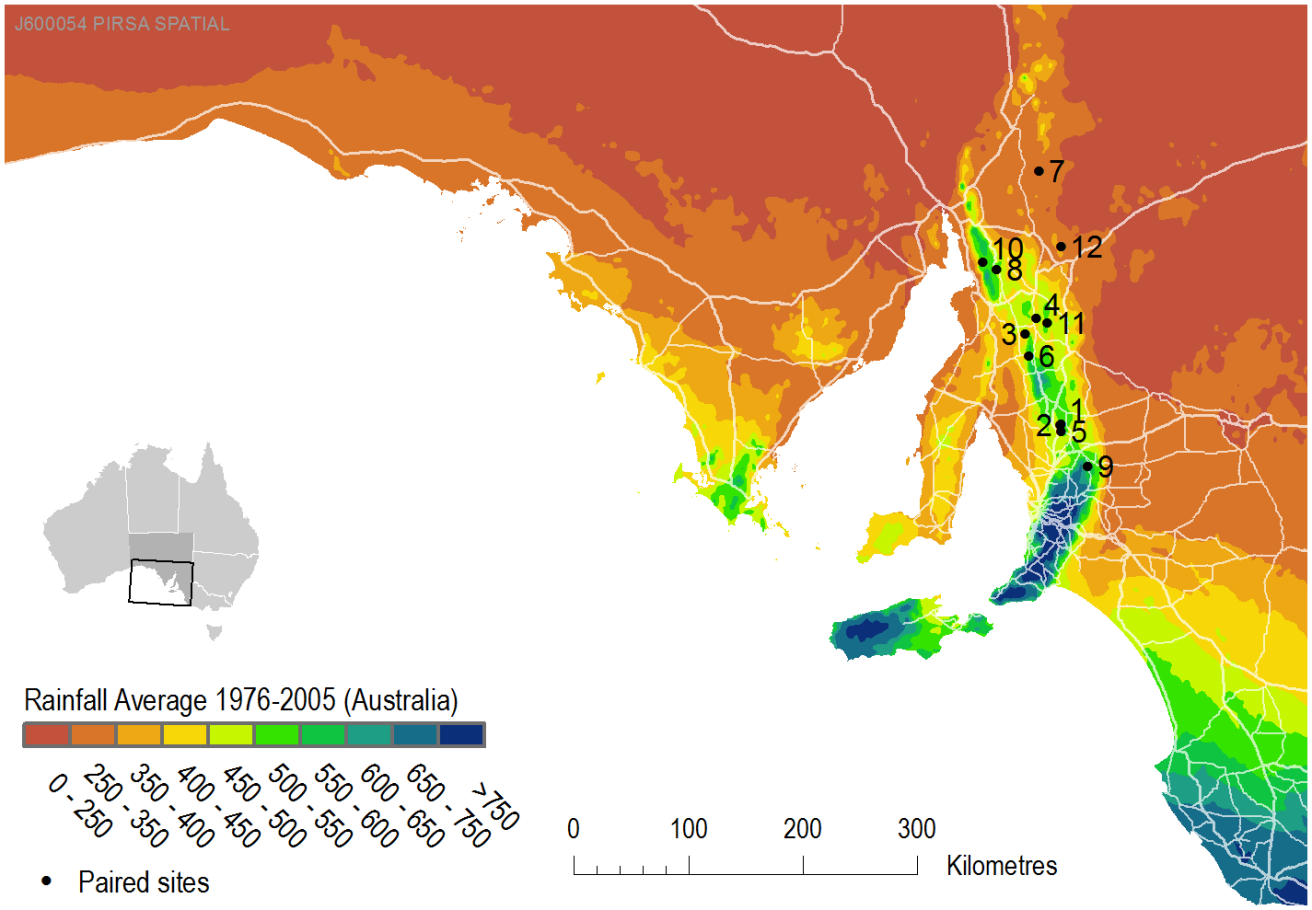
Site locations plotted over a rainfall map of South Australia.

Twelve rotationally grazed paddocks were selected based on two criteria: 1) landholders had been managing livestock in a consistent way for a period of at least seven years (preferably 10 years); and 2) a nearby continuously grazed paddock could also be identified that was located on the same soil type and landscape position (Fig 1). The distance between pairs ranged from across a fence line up to 10 km in one case to find a paired continuously grazed paddock with the same soil properties. The 7–10 year minimum time period should allow for pasture composition to stabilize as species sensitive to the change in regime could have been lost from the species assemblage following a change in grazing management [34]. Rotationally grazed paddocks ranged in area from 6 to 79 ha, while continuously grazed paddocks ranged from 17 to 2670 ha. Wherever possible a site with native remnant vegetation with no actively managed grazing on the same soil type was also sampled. A total of four native vegetation sites were sampled. Rotational grazing sites #1 and #2 share a common continuously grazed site and native remnant vegetation site.

Paddock information and management histories were collected for all paddocks sampled including livestock grazing, cultivation and fertilizer management, fire history and other grazing impacts [35]. The management history is based on landholder records, observations and discussions about their current and previous management of individual paddocks where possible. Permission was obtained in writing from all of the private landowners prior to field work. For each site, we also collected climate data for the period 1980 to 2011 for each site were extracted from the SILO database [36] and various topographic attributes were derived from the 1 arc-second resolution Smoothed Digital Elevation Model of Australia [37]. For climate, the 5 year and 30 year mean daily temperature (°C), total rainfall (mm) and average vapour pressure deficit (kPa) for winter, summer and annual periods were considered. For topographic attributes, elevation, aspect, slope, plan curvature, profile curvature and derived indices related to wetness (topographic wetness index, TWI; topographic position index, TPI) were recorded [38].

Pasture productivity was estimated using the normalized difference vegetation index (NDVI). Mean monthly NDVI values were derived from the 250 m resolution MODIS NDVI (MOD13Q1) 16-day composite mosaics for Australia [39], [40], [41]. The 16-day composites were aggregated to monthly values, calculated as the average of the 16-day periods that overlap with a month, weighted by the number of days of the overlap. Monthly NDVI was then summed to give an annual NDVI value for each of the years since conversion to rotational grazing for each pair of sites. Estimating productivity as the sum of monthly NDVI will underestimate true productivity because of biomass removal during defoliation events. Grazing pressure was either equal or slightly greater in the rotationally grazed pair at each site [35], so there is a chance that our NDVI surrogate for productivity is slightly biased towards the continuously grazed paddock in each pair.

### 2.2. Field sampling

A preliminary survey of one rotationally grazed paddock where 120 cores were collected in a nested geospatial design was used to guide the choice of field sampling design [42]. This preliminary investigation found that it was most important to capture differences in soil types which were associated with different geomorphic surfaces. Upland residual surfaces and upper slopes often contained Calcarosols while Red Chromosols were typically encountered on lower slopes and alluvial surfaces. Based on these findings we chose a sampling design where each paddock was divided into six strata and within each strata four soil cores (inner diameter = 54 mm) were randomly collected using a hydraulic trailer mounted corer and bulked by depth interval (0–10, 10–20, 20–30 and 30–50 cm). Where paddocks were larger than approximately 10 ha, a 10 ha sub-region was selected for sampling with the selection criteria being to best pair the soil properties between two management categories. Strata were determined on a paddock by paddock basis based on expert opinion of the landform. If obvious different geomorphic surfaces and hence potential differences in soil type were present, then strata were divided amongst different landforms. Where paddocks looked superficially similar, the paddock was divided into six strips of roughly equal dimensions. Samples for determination of bulk density (BD) for the same depth intervals were collected separately using a set of stacked rings (middle ring was 80 mm long with an I.D. = 100 mm) with a slide hammer at one location within each of the six strata at each paddock. All soil sampling occurred between June and August 2013 when paddocks were being rested from grazing.

### 2.3. Laboratory methodologies

Field moist samples were weighed, dried at 40°C for 48 hrs, re-weighed and then passed through a 2 mm sieve. The mass of gravel retained on the sieve was recorded. Subsamples of the air-dried fine earth fraction were further dried for 24 hrs at 105°C to determine the residual moisture content and to correct all data to an oven-dry basis. The remaining fine earth fraction was quantitatively split using a riffle box down to a 10 g sample which was then finely ground on a Retsch MM400 Mixer Mill (Retsch GmbH, Germany). Bulk density samples were dried at 105°C for 48 hrs before weighing.

All soil samples were analyzed for total carbon (TC) and total nitrogen (TN) by high temperature oxidative combustion on a LECO Trumac CN analyser (LECO Corp., MI, USA). Samples that were found to contain carbonates upon application of 4 M hydrochloric acid (HCl) were then pre-treated with 6% sulphurous acid (H_2_SO_3_) before analysis for TC (method 6B3 [43]). Where carbonates were not present total organic carbon (TOC) equaled TC; however, when carbonates were present TOC was taken as the carbon concentration after H_2_SO_3_ pretreatment and inorganic carbon (IC) was calculated as the difference between TC and TOC.

Diffuse reflectance mid-infrared (MIR) spectra were obtained on all samples following the sample protocols presented in Baldock et al. [44] on a Nicolet 6700 FTIR spectrometer (Thermo Fisher Scientific Inc., MA, USA) equipped with a KBr beam splitter, a DTGS detector and an automated diffuse reflectance accessory (AutoDiff, Pike Technologies, WI, USA). The allocation of TOC to biologically significant carbon fractions (particulate OC (POC), humus OC (HOC) and resistant OC (ROC) [45]) was estimated using a partial least squares regression (PLSR) model developed by Baldock et al. [44].

Electrical conductivity (EC) and pH were measured on 120 samples (one core from each paddock) in a 1:5 soil-to-water suspension and MIR-PLSR regressions were developed to predict these values for the remaining samples. The best fit PLSR model for pH included 6 factors with an R^2^ = 0.76 and RMSE = 0.439. Electrical conductivity did not predict as well because there was generally a small range of values across these sites (R^2^ = 0.61, RMSE = 0.090). Clay content was predicted using algorithms from an Australian soil spectral library [46].

### 2.4. Data analysis

Given the diversity of management data collected, we sought to understand the most important parameters differentiating rotational grazing from continuous grazing. This was accomplished through multivariate analysis of these data using the PRIMER 6 and PERMANOVA+ software packages (Primer-E Ltd., Plymouth, UK). First the continuous data on rest periods per annum, rest days per period, stocking rate and grazing days per annum were normalized and a resemblance matrix using Euclidean distance between samples was generated. A distance-based linear model using the BEST routine [47] then identified the most important variables to describe the management data. A permutational multivariate analysis of variance (PERMANOVA [48]) was also run on the reduced set of data to test for significant difference between the management practices. Principal coordinates [49] and cluster [50] analyses were also performed on this reduced dataset to visualize the data and find significant grouping.

In order to determine if productivity, as assessed by annual summed NDVI, in the rotationally grazed sites was increasing over time relative to the continuously grazed pairs, we calculated the log response ratio (LRR_NDVI_) of NDVI as ln(NDVI_rotational_/NDVI_continuous_) for each year since establishment of rotational grazing and applied linear regression analysis to examine if there had been a significant increase in productivity under rotational grazing.

Soil organic carbon stocks for each horizon (SOC_*h*_, Mg C ha^-1^) were calculated as 10 x thickness (m) x BD (Mg d.w. m^-3^) x (1 –gravel) x TOC (kg C Mg d.w.^-1^). In order to minimize any potential difference in SOC stocks due to differences in sampling volume to a fixed depth, we converted all SOC stocks to an equivalent mass basis (SOC_eq_) [51]. We chose the average fine fraction mass to 30 cm across all sample sites (4251 ± 271 Mg soil ha^-1^). For cores with < 4251 Mg ha^-1^ in the 0–30 cm layer, SOC_eq_ was calculated as SOC_0-10_ + SOC_10-20_ + fraction of SOC_20-30_ that would then equal the average soil mass. If the average mass was greater than average, then SOC_eq_ was calculated as SOC_0-10_ + SOC_10-20_ + SOC_20-30_ + fraction of SOC_30-50_ to equal the average soil mass.

Mean SOC_eq_, POC_eq_ and C:N ratio data from each site were analyzed using a one-way analysis of variance (ANOVA) with management category (rotational (n = 12), continuous (n = 11), native remnant (n = 4)) as the main factor. Data were square root transformed prior to analysis to normalize variance.

At the regional scale, climate and soil properties are often the major factors determining the SOC content of a given soil [52]; as such, we have attempted to minimize these covariates in both the experimental design and in subsequent statistical analyses. This study was set up to assess pairs of grazing management strategies as to minimize covariates at each location. Focusing only on the grazed paddocks, we calculated log response ratios (LRR_SOC_) at each site as ln(SOC_rotational_/SOC_continuous_) following [53].

To better understand the dominant drivers of the observed patterns, correlation and multiple linear regression analyses were performed with the SOC_eq_ and LRR data. After screening and removing redundant results based on the Pearson Product Moment Correlations, the Best Subsets Regression routine in SigmaPlot 12.0 (Systat Software Inc., San Jose, USA) with adjusted R^2^ as the selection criteria was used to build the best multiple linear regression model. The main factors that we considered in these analyses could be grouped into four categories: climatic, topographic, soil properties and property management. In a second approach to minimize the overriding climate and soil controls on SOC, the residuals, SOC_residual_, of the regression between SOC_eq_ and climate/soil properties were further regressed against management data.

## Results and Discussion

### 3.1. Management data

**Fig 2:**
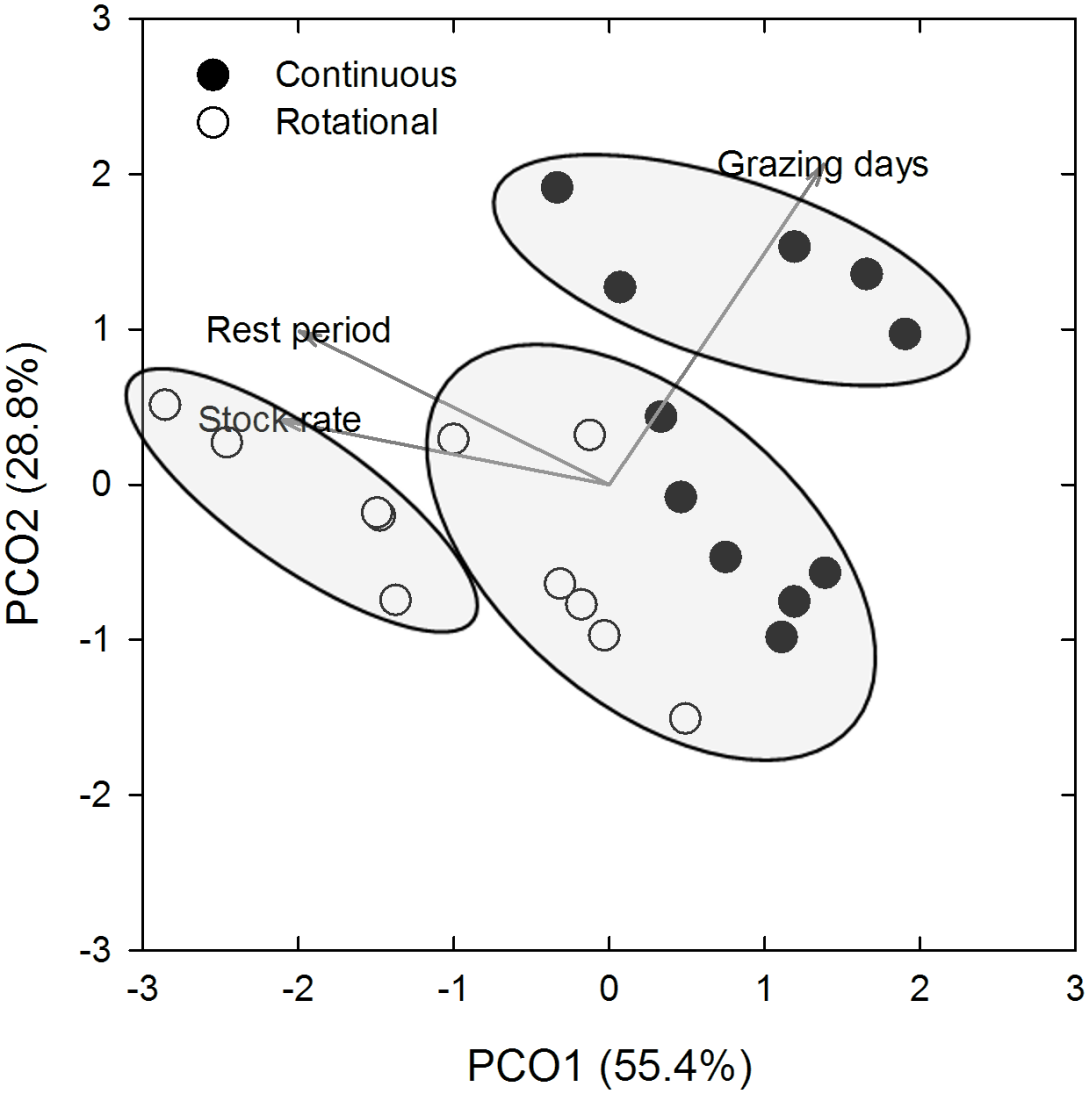
Principal coordinates analysis of management data. Influence of grazing days, stocking rate and rest periods are given in grey vectors. PC1 explained 55.4% of the variance and PC2 explained an additional 28.2%. Clusters are shown in black to indicate most similar groupings.

This study was designed under the premise that there are significant differences between rotational and continuous grazing management practices; however, the reality is that there are a myriad of implementations of each of these management practices. Multivariate analysis of grazing management variables, rest periods (days of rest per year), stocking rate and grazing days, found that there were indeed highly significant differences between rotational and continuous stocking practices (one-way PERMANOVA, pseudo-F = 11.14, P < 0.001). In a principal coordinates analysis (PCO) of these same data, it was clear that the two categories of grazing management separate out but that there was variation in grazing variables (e.g. stocking rate, length of rest period and number of grazing days/annum) across this management spectrum (Fig 2). A cluster analysis demonstrated that there is a group of farmers who practice a more continuous form of rotational grazing (e.g., lower stocking rate and more grazing days than other rotational graziers) or a more rotational form of continuous stocking (e.g., multiple or longer rest periods). The fact that farmers implement a range of practices under a generic management category will not come as a surprise to the farmers themselves, but this is a critically important point in interpreting data from comparative studies such as this one. The variability introduced due to this gradient in management may preclude finding a treatment effect when lumping data into broad management categories.

### 3.2. Pasture productivity

**Fig 3:**
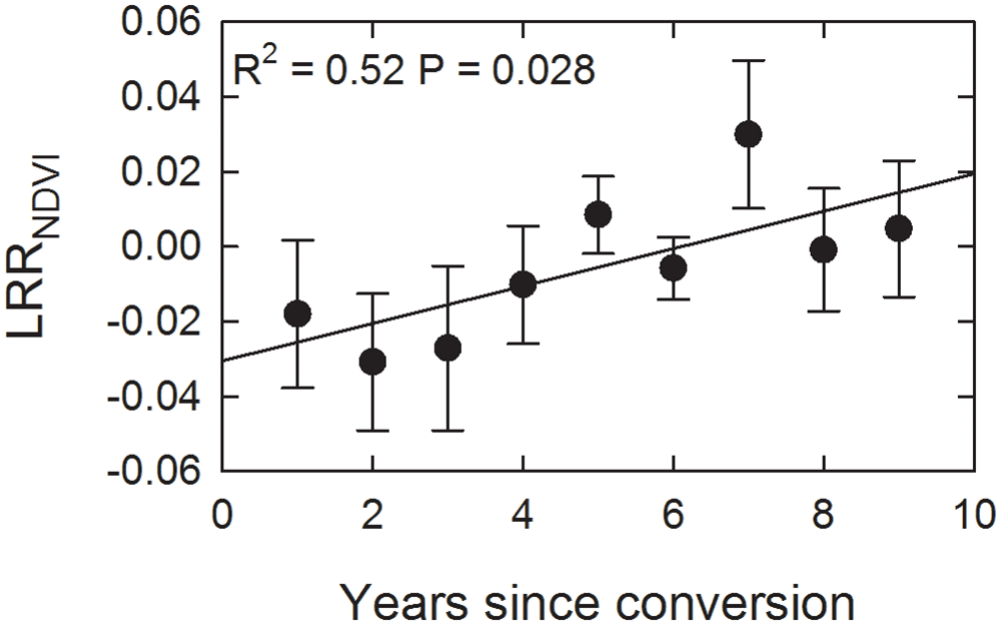
Trend of increasing log response ratio between NDVI of rotationally grazed versus continuously grazed paired paddocks (LRR_NDVI_) with time since conversion of management. Only the first 9 years since conversion were included because only 5 sites were under rotational management for longer than that time. Error bars represent 1 standard error.

Pasture productivity, as assessed by the mean annual summed NDVI over the number of years of rotational grazing implementation, was no different between the two management classes (NDVI_rotational_ = 5.46 ± 0.82, NDVI_continuous_ = 5.55 ± 0.95, ANOVA F = 0.055, P = 0.82). It is important to recognize that NDVI may be underestimating the true productivity of pastures with correlation coefficients between NDVI and measured grass yields typically being between 0.6 and 0.8 [54]. This underestimation may be greatest under high grazing pressure, thus potentially masking real differences between grazing management. While the overall means did not show any patterns, when plotting mean LRR_NDVI_ across time, there was a significant positive trend (Fig 3) suggesting that under rotational grazing productivity can increase relative to continuous grazing.

Productivity has been found to increase through the implementation of rotational grazing, from both a pasture and livestock production perspective, although the experimental evidence is mixed [20]. Improvements in pasture productivity with rotational grazing have been attributed to enhanced growth and survival of plant species [12], [13], increased herbage production [10], [18], [55], and greater perennial grass content and basal area through regeneration of plants [11], [19], [20]. These improvements in the pasture base can then contribute to improved animal production through increased stocking rates [16].

### 3.3. Soil organic carbon trends

Across all sampled paddocks, there were no significant differences for SOC_eq_ or SOC_0-10_ between management categories (Table 1). Mean SOC_eq_ stocks were 48.9 ± 4.5, 49.4 ± 5.5 and 47.7 ± 11.7 Mg C ha^-1^ (± 1 s.e.m.) for rotationally grazed, continuously grazed and remnant native vegetation, respectively. While total stocks did not vary, the proportion of SOC found as particulate organic carbon (POC) and the C/N ratios were found to vary significantly, with the remnant native vegetation sites having a greater proportion of POC and higher C/N ratios than either of the grazing categories (Table 1).

**Table 1:** Selected soil organic matter properties summarized by grazing management category (mean ± 1 s.e.m. presented). For properties with significant differences amongst grazing management categories (rotational and continuous grazing), pairs that differ from each other are given with different letters.

Management	n	SOC_eq_	SOC_0-10_	SOC_0-10_/ SOC_eq_	POC_eq_	POC/SOC	C/N
		(tC ha^-1^)	(tC ha^-1^)	(%)	(tC ha^-1^)	(%)	
Rotational	12	48.9 ± 4.5	24.4 ± 2.4	49.9 ± 2.2	7.0 ± 1.6	13.5 ± 2.6^a^	9.9 ± 0.6^a^
Continuous	11	49.4 ± 5.5	26.3 ± 2.8	52.2 ± 2.6	7.6 ± 1.8	14.2 ± 2.9^a^	10.3 ± 0.8^a^
Native	4	47.7 ± 11.8	28.1 ± 8.8	48.3 ± 5.9	9.9 ± 3.9	20.6 ± 5.0^b^	12.2 ± 1.4^b^
ANOVA summary							
F statistic		0.077	1.419	2.209	2.098	5.460	8.144
P value		0.926	0.245	0.113	0.126	0.005	0.001

*abbreviations: SOC_eq_ = soil organic carbon stock to equivalent mass; SOC_0-10_ = C stock in top 10 cm; POC_eq_ = particulate organic carbon stock in equivalent mass; C/N = carbon to nitrogen ratio.

**Fig 4:**
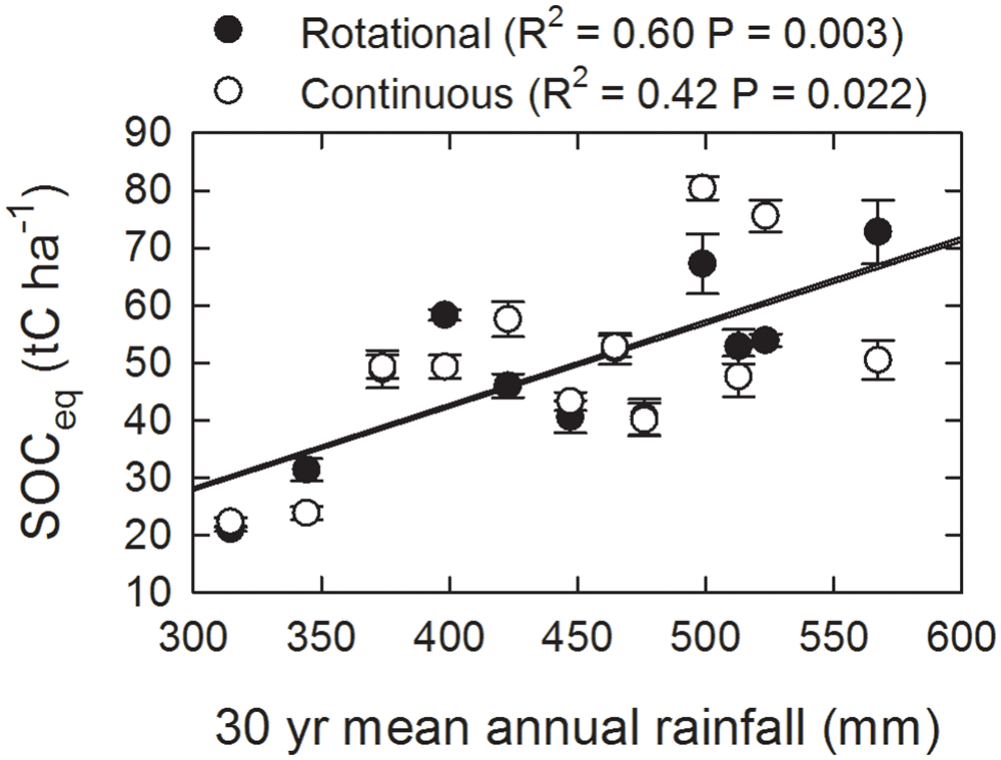
Relationship between mean annual rainfall and SOC_eq_ for rotationally and continuously grazed paired sites.

Given the large climatic gradients especially in rainfall (Fig 1) across this region, we expected that we would find large regional differences in SOC stocks. Indeed, there were strong correlations between SOC stocks and temperature, rainfall (Fig 4), pasture productivity (NDVI) and topographic attributes related to wetness (Table 2). Measured or predicted soil properties (pH, EC and clay) did not correlate well with SOC stocks. There was no correlation between temperature and rainfall, but NDVI was correlated with climatic indices (NDVI v. winter temperature, R = 0.47, P = 0.021; NDVI v. annual rainfall, R = 0.52, P = 0.009; NDVI v. annual vapour pressure deficit, R = -0.72, P < 0.001). Slope, topographic wetness index (TWI) and topographic position index (TPI) were all correlated (TWI v. slope, R = 0.67, P < 0.001; TWI v. TPI, R = -0.68, P < 0.001). When used in a multiple linear regression, mean winter temperature and mean annual rainfall could explain 61% of the variance in SOC (R^2^ = 0.61, ANOVA F = 19.99, P < 0.0001). These findings are consistent with well established ecological principles that climate is a master variable controlling SOC stocks at regional scales due to its overriding influence on primary productivity and microbial activity [22].

**Table 2:** Pearson’s correlation coefficients between potential SOC covariates and SOC_eq_ stocks (n = 27 for climatic and topographic variables, n = 23 for NDVI and management variables) and LRR_SOC_ (n = 12).

	SOC_eq_		LRR_SOC_	
Variable	R	P value	R	P value
30 yr mean Apr-Oct temperature	0.50	0.01	-0.43	0.16
30 yr mean annual rainfall	0.61	0.00	-0.09	0.79
30 yr mean Nov-March VPD	-0.41	0.02	-0.13	0.67
NDVI	0.59	0.00	n.a.^c^	
Slope	0.41	0.02	n.a.	
Topographic wetness index (TWI)	-0.53	0.00	n.a.	
Stocking rate	-0.02	0.91	-0.07	0.84
Rest days per year	0.22	0.30	0.31	0.33
Grazing days	-0.05	0.79	0.44	0.15
Management PCO1^a^	-0.04	0.85	-0.14	0.66
Management PCO2^a^	-0.02	0.93	0.47	0.12
Management distance b/t pairs^b^	n.a.		0.26	0.42

^a^Principal coordinate axes from Fig 2.

^b^Euclidean distance between rotational and continuously grazed pairs in principal coordinate space (Fig 2).

^c^n.a. = not an appropriate comparison.

When only the grazed paddocks were considered, TWI and rest periods (average # of rest periods in a year) added additional explanatory power to the regression (R^2^ = 0.72) but each variable only explained an additional 3–6% of the variance in SOC_eq_. Due to the multicollinearity of NDVI with climatic indices, inclusion of NDVI in the multiple linear regression did not improve the prediction of SOC_eq_. We also compared the management data to the residuals from the multiple linear regression with mean winter temperature and mean annual rainfall and found that rest periods (days of rest per year) was the only single variable that could explain a significant fraction of the residual variance (Pearson’s R = 0.44, P = 0.04). In a multiple linear regression, rest periods, stocking rate and grazing days could explain 58% of the variance in the residuals of SOC_eq_ (ANOVA F = 7.86, P = 0.002) although the only significant term (P < 0.05) was rest periods.

SOC_residuals_ = -4.21 + (2.54 × rest periods) + (0.23 × stocking rate)–(0.31 × grazing days). Inspection of this equation reveals that both rest periods and stocking rate, the two management variables that were positively associated with rotational grazing practices (Fig 2) positively influenced SOC_residuals_ suggesting that rotational grazing may be leading to marginal improvements in SOC.

### 3.4. Paired site SOC analysis

**Fig 5:**
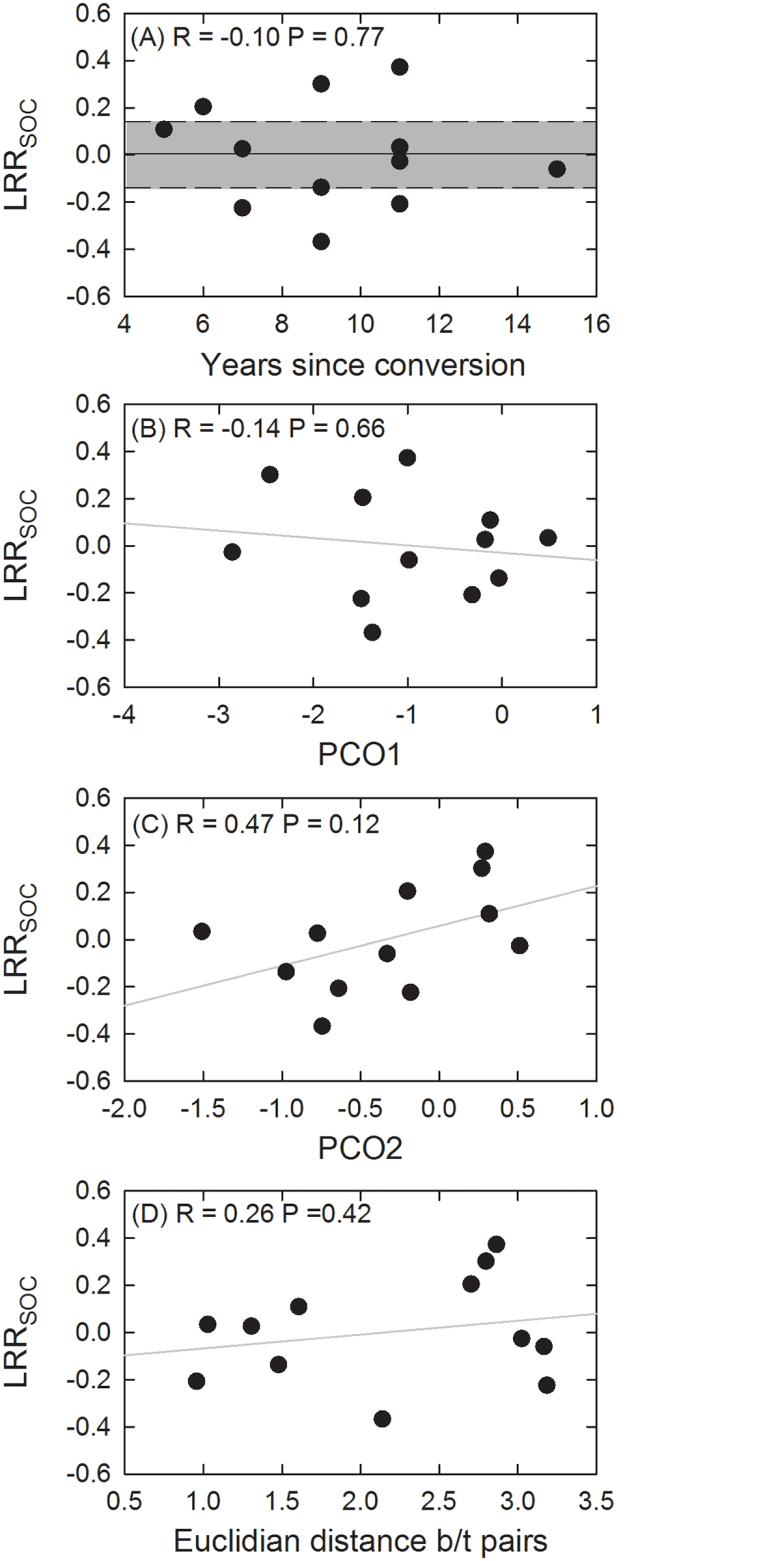
Log response ratio of SOC_eq_ (LRR_SOC_) between pairs plotted against years under rotational management (A), principal coordinate axis 1 and 2 (B & C), and the Euclidean distance between management pairs (D). Linear trends with Pearson correlation coefficients given on each plot expect in (A) where the mean LRR_SOC_ and 95% confidence intervals are plotted.

A paired site approach was specifically adopted in this study to minimize the influence of climatic controls on SOC stocks in the interpretation of potential changes due to grazing management. Across all 12 sites, the mean LRR_SOC_ was 0.004 ± 0.058 (1 s.e.m.). While there were no significant correlations between LRR_SOC_ and any of the soil, climate, topographic or management variables (Table 2), management variables (i.e., PCO2 from the management ordination) again could explain about 20% of the variability in the LRR_SOC_ data (Fig 5C). Given that the variable grazing days was most strongly correlated with PCO2 (Fig 2), this finding suggests that amongst the rotationally grazed sites the sites with the strongest grazing pressure showed the greatest SOC response.

### 3.5. Management effects on SOC

In this study, despite significant differences in management practices (Fig 2), which has led to minor shifts in pasture productivity (Fig 3), there were no clear trends in SOC stocks (Fig 5A). Management factors were able to explain 58% of the residual variance in SOC_eq_ after taking away the overriding trends with climate. This value of 58% equates to 22% of the total variance in SOC_eq_ across our study region. Interestingly, the second principal coordinate axis (PCO2) could also explain 22% of the variance in LRR_SOC_ (Fig 5C). Given all the sources of uncertainty in measuring differences in SOC stocks, we are cautious to make a strong case out of this limited finding and feel the preponderance of evidence in this study suggests that implementation of rotational grazing is only having a small positive effect on SOC stocks. A finding of no significant management effect on SOC stocks is still an important finding in the context of managing land for greenhouse gas abatement.

The scientific literature is decidedly mixed with some studies showing positive SOC responses to rotational grazing (e.g. [56], [57]), many showing no significant difference (e.g. [28], [29]) and a few suggesting a slight negative SOC response (e.g. [27], [58]). Most of the positive responses of SOC to adoption of rotational grazing seem to be located in more mesic rangelands because of the shorter recovery times needed for the vegetation between defoliations [10]. Within Australia, field studies have failed to find a significant grazing management effect on SOC stocks. Wilson and Lonergan [29] found no difference in SOC stocks in the 0–30 cm layer between similar styles of rotational and continuous grazing of improved pastures in the central Tablelands, NSW. Chan et al. [28] found significantly higher SOC stocks in native and introduced pastures which had been improved with phosphorus often associated with the addition of legumes (grazing management controlled for), but no significant differences in SOC between rotational and continuous grazing management in central and southern NSW. Allen et al. [27] found a small negative influence of stocking rate on SOC stocks but no significant differences between grazing management styles in the rangelands of Queensland. Despite some uncertainties in the absolute quality of the pairings suggested by the SOC and land management data, our finding of no significant impact of rotational grazing on SOC stocks relative to continuous grazing is in accord with similar work in other regions of Australia and the globe.

In a recent synthesis of rotational grazing experimental research, Briske et al. [21] concluded that “continued advocacy for rotational grazing as a superior strategy of grazing on rangelands is founded on perception and anecdotal interpretations, rather than an objective assessment of the vast experimental evidence.” While the Briske et al. [21] review did not focus on soil properties, only 3 of 23 studies found significantly higher plant production in rotational compared to continuously grazed treatments. Given that gains in plant productivity and root turnover to the soil are the most likely reasons that rotational grazing may lead to greater SOC stocks, the fact that so few studies have found gains in production suggest that gains in SOC may be the exception rather than the rule.

Detecting change in SOC has several added layers of complexity. As seen in this study and others [28], [29], the inherent variability in SOC within paddocks and across small regions makes detecting small but real improvements in SOC difficult. Additionally, an important finding from this study was that there was a large range of grazing intensities within each management category (Fig 2) and treating all sites as equal within each of the two main categories may be masking real differences in the dependent variable [59].

## Conclusions

The evidence from this study suggests that in the short to medium term (< 15 years) measurable gains in SOC with adoption of rotational grazing in similar eco-climatic zones should not be expected to be observed. The data analysis presented here provided limited support to the hypothesis that rotational grazing management can positively change SOC stocks, but detecting this small signal relative to the large variability in SOC is exceedingly difficult. While we did not detect positive trends in SOC over time, NDVI data indicated that plant production was increasing slightly under rotational relative to continuous grazing. This finding of increasing productivity suggests that given enough time observable gains in SOC may be found.
